# Primary central nervous system lymphomas express immunohistochemical factors of autophagy

**DOI:** 10.1038/s41598-021-01693-6

**Published:** 2021-11-15

**Authors:** Georgia Karpathiou, Silvia-Maria Babiuc, Florian Camy, Elise Ferrand, Alexandra Papoudou-Bai, Jean Marc Dumollard, Jerome Cornillon, Michel Peoc’h

**Affiliations:** 1 Pathology Department, University Hospital of Saint-Etienne, 42055 CEDEX2 St-Etienne, France; 2 Hematology and Cell Therapy Department, Lucien Neuwirth Cancer Institute, Saint Etienne, France; 3 Pathology Department, University Hospital of Ioannina, Ioannina, Greece

**Keywords:** Cancer, Immunology, Molecular biology, Neuroscience, Pathogenesis

## Abstract

Primary central nervous system lymphoma (PCNSL) is an aggressive and rare disease. Autophagy is a catabolic mechanism boosting various tumors, including lymphomas; its inhibition is thus a promising therapeutic target. Its presence has never been studied in PCNSLs. We conducted a retrospective immunohistochemical study of 25 PCNSLs for LC3B, p62, and M6PR, comparing it with clinicopathological characteristics. Fourteen (56%) and eleven (44%) PCNSLs were of low and high LC3B expression, respectively. p62 expression was present in most tumors (n = 21, 84%). M6PR was present in all tumors, with 14 (56%) and 11 (44%) cases being of low and high M6PR expression, respectively. LC3B expression was correlated with the performance status (PS) (p = 0.04). No association was found with other clinical parameters, such as deep structure invasion, multiple lesions, complete response, and recurrence after response. p62 showed a strong positive association with MUM1 expression (p = 0.0005). M6PR expression showed a positive correlation (p = 0.04) with PD-L1 expression. No association was found with p53, Ki67, CD8, BCL2, BCL6, or double MYC/BLC2 co-expressors. No association of LC3B, p62, and M6PR expression with survival was found. Our findings provide evidence for the possible presence of autophagic markers in PCNSLs and, thus, for possible treatment targets.

## Introduction

Primary diffuse large B-cell lymphoma (DLBCL) of the central nervous system (CNS) is an aggressive and rare lymphoma of the brain, spinal cord, leptomeninges, or eye^1^. It accounts for < 1% of all non-Hodgkin lymphomas and around 3% of all brain tumors, with an overall annual incidence rate of 0.47/100,000 population^1^. Patients with this rare lymphoma type show a median overall survival time of 37 months, with 1-, 2-, and 5-year survival rates of 76%, 63%, and 37%, respectively^2^.

Autophagy is a mechanism leading to the isolation and degradation of intracellular damaged constituents, occurring at basal levels in healthy cells^3^. Enhanced autophagy seems to aid tumor cells by providing them with recycled metabolic nutrients and by eliminating cellular debris^3^. Thus, the inhibition of autophagy in various tumor types, including lymphomas, may be a therapeutic target^3^. The autophagic machinery involves several proteins that create a vesicle called an autophagosome, isolating material inside the cytoplasm which is then fused with lysosomes for degradation^4,5^. There are autophagy receptors that bind to cargos and lead them to the autophagic vesicles by interacting with LC3—the principal component of the autophagosome—with the best studied being sequestosome 1 (SQSTM1)/p62^4,5^. p62, along with the autophagic cargo, is degraded; thus, reduced levels of p62 are considered a surrogate marker of activated autophagy^4,5^. Thus, the two molecules LC3 and p62 are often used together to assess autophagy: LC3 as a surrogate marker of autophagic vesicles and p62 as a surrogate marker of autophagic degradation and, thus, of the completed pathway^5^. The cation-independent mannose-6-phosphate receptor (M6PR) is the prototypical lysosome-targeting receptor and the main endosomal marker associated with the autophagic machinery^6^. To the best of our knowledge, the presence of autophagy has never been studied in PCNSL. Thus, the aim of this study was to investigate the possible presence of autophagic markers in PCNSLs and to compare it with the clinicopathological prognostic features of this disease.

## Materials and methods

In this single-center retrospective cohort study, we included all consecutive patients with histologically confirmed primary CNS DLBCL who were diagnosed between 2007 and 2015 and categorized according to the 2017 World Health Organization (WHO) criteria^1^ by a specialized hematopathologist (MP). Patients with systemic disease prior to or synchronous with the PCNSL (n = 73), with insufficient data for formally excluding systemic disease (n = 46), or with insufficient histologic material (n = 16) were excluded, leading to a final cohort of 25 patients.

The clinicopathological characteristics of this cohort have been previously published^7^: the Memorial Sloan Kettering Cancer Center (MSKCC) score was used as a prognostic model^2^. According to this model, patients ≤ 50 years of age (group A) have the best prognosis, followed by patients older than 50 years with a KPS of ≥ 70 (group B), and finally, patients older than 50 years with a KPS of < 70 (group C) show the worst prognosis^2^. Invasion of deep brain structures, defined as the periventricular regions, basal ganglia, corpus callosum, brainstem, and/or cerebellum, and multiplicity of the lesions were also recorded^8^.

Baseline immunohistochemical features with prognostic features according to previous studies were also recorded: The immunohistochemical classification scheme developed by Hans et al. for systemic DLBCL subdividing tumors into germinal center B-cell-like (GCB) and non-germinal-center-like (ABC-activated B cell) based on the expression patterns for CD10, BCL6, and MUM1 was used^9^. BCL2 and MYC expression levels (double co-expressors were defined by BCL2 ≥ 70% and MYC ≥ 30% of tumor cells^10^) were also assessed due to their prognostic significance in this tumor type^10,11^. Similarly, given the importance of the immune microenvironment in PCNSLs^12^, PD-L1 (22C3, Dako Agilent) and CD8 expression were analyzed: the percentage of PD-L1 membranous staining of tumor cells and the percentage of CD8 tumor-infiltrating lymphocytes were recorded^13^.

Immunohistochemistry for the autophagic markers was performed on formalin-fixed, paraffin-embedded 4 μm thick full tumor sections using an automated staining system (OMNIS, Dako-Agilent, Santa Clara, CA, USA). The primary antibodies used were: LC3B (Rabbit monoclonal, ab192890, abcam, dilution 1/1000, pH 6, 20 min), SQSTM1/p62 (Rabbit monoclonal, ab109012, abcam, dilution 1/2000, pH 6, 20 min), and M6PR (cation independent) (Rabbit monoclonal, ab124767, abcam, dilution 1/2000, pH 6, 20 min). Positive immunoreactions were visualized using 3,3′-diaminobenzidine as the chromogenic substrate. The antibodies were initially tested in a large variety of normal and neoplastic tissues to decide the best immunohistochemical protocol giving no background staining and a range of staining intensities^14^. Thereafter, nerve fibers and normal tonsillar tissue were used as positive controls for LC3B and p62^15^, respectively, as well as tonsillar tissue for M6PR, while omission of the primary antibody was used as a negative control^14^. Normal brain tissue also served as an internal positive control for LC3B and p62 in the current series. The intensity and percentage of the immunohistochemical staining of each case were recorded. LC3B^15^ and M6PR staining was presented as cytoplasmic punctae and according to the density of dots per cell; it was recorded as negative (intensity score 0, no staining or ≤ 10 dots per cell), mild (intensity score 1, 11–20 dots per cell), moderate (intensity score 2, > 20 dots per cell without clusters), or strong (intensity score 3, > 20 dots per cell with clusters). The intensity of p62 staining was recorded as negative, weak, moderate, or strong^16^. The percentage of cells positive for LC3B and M6PR was recorded from 0 to 100% and presented as the H score (percentage of positive cells multiplied by the intensity). Staining for p62 was uniform across all the cells of the same tumor, and both a four-tiered system (negative, weak, moderate, and strong) and a two-tiered system (negative/mild/moderate vs. strong^16^) were used for statistical analyses. We further combined the immunoexpressions of LC3B and p62 into basal autophagy characterized by low LC3B and low or high p62, activated autophagy showing high LC3B and low p62, and activated but then blocked autophagy, showing high LC3B and high p62 expression^5,15^.

Data were analyzed using the StatView software (Abacus Concepts, Berkley, California): we used the x^2^ test to explore the relationship between two groups (categorical data), factorial analysis of variance (ANOVA) to explore the relationship between a factor (categorical data) and a continuous parameter, and simple regression analysis between two continuous parameters. We considered immunohistochemical factors both as continuous variables and as ordinal variables, using their mean value as the cut-off level. Survival probability was estimated via Kaplan–Meier analysis with log-rank product limit estimation. For all analyses, statistical significance was indicated at a *p* value of < 0.05.

## Results

### Clinical data

The current cohort (Table 1), as also previously described^7^, included 12 (48%) female and 13 (52%) male patients with a median age at diagnosis of 66 years (35–85 years). None of the patients showed HIV infection. Performance status was ≥ 70 in 12 (48%) patients, and the MSKCC score was of group A in 3 patients (12%), group B in 10 (40%) patients, and group C in 12 (48%). The invasion of deep structures and multiplicity were seen in 16 (64%) and 9 (36%) patients, respectively. Complete response was seen in 11 patients (47.8%), and recurrence occurred in 4 of them (36.3%). Fourteen patients died of the disease during follow-up, which ranged from 1 to 108 months (median 24 months). Overall survival (log-rank) ranged from 1 to 108 months with a median of 36 months. These demographic data are generally in line with those reported in the literature^2,8^.

**Table 1:** Clinical and immunohistochemical features of the lymphomas studied.

Parameter	n, %
**Age**	
Range	35–85
Mean ± SD	63.7 ± 12.6
Median	66
≤ 50 years	3, 12%
> 50 years	22, 88%
**Sex**	
Female	12, 48%
Male	13, 52%
**Performance status**	
≥ 70	12, 48%
< 70	13, 52%
**Memorial Sloan Kettering score**	
A	3, 12%
B	10, 40%
C	12, 48%
**Invasion of deep brain structures**	
Yes	16, 64%
No	9, 36%
**Multiple lesions**	
Yes	9, 36%
No	16, 64%
**Complete response (n = 23)**	
Yes	11, 47.8%
No	12, 52.2%
**Recurrence after complete response (n = 11)**	
Yes	4, 36.3%
No	7, 63.6%
**MUM1 (n = 24)**	
Yes	17, 70.8%
No	7, 29.2%
**BCL2 (n = 23)**	
Yes	15, 65.2%
No	8, 34.8%
**CD10 (n = 25)**	
Yes	0
No	25, 100%
**BCL6 (n = 22)**	
Yes	16, 72.7%
No	6, 27.3%
**MYC (%)**	
Range	0–80
Mean	15.4 ± 18
Median	10
**p53 (%)**	
Range	0–100
Mean	26 ± 30.2
Median	10
**CD8 (%)**	
Range	1–50
Mean	11.2 ± 11.9
Median	10
**PD-L1 (%)**	
Range	0–90
Mean	27.6 ± 33.4
Median	10
**Ki67**	
Range	15–80
Mean	46.5 ± 13.6
Median	50
**Germinal center (GC)-type lymphoma (n = 24)**	
GC type	6, 25%
ABC type	18, 75%
**Double co-expressors MYC/BCL2**	
Yes	3, 12%
No	22, 88%

*n* = 25 unless otherwise specified.

### Immunohistochemical findings

**Figure 1:**
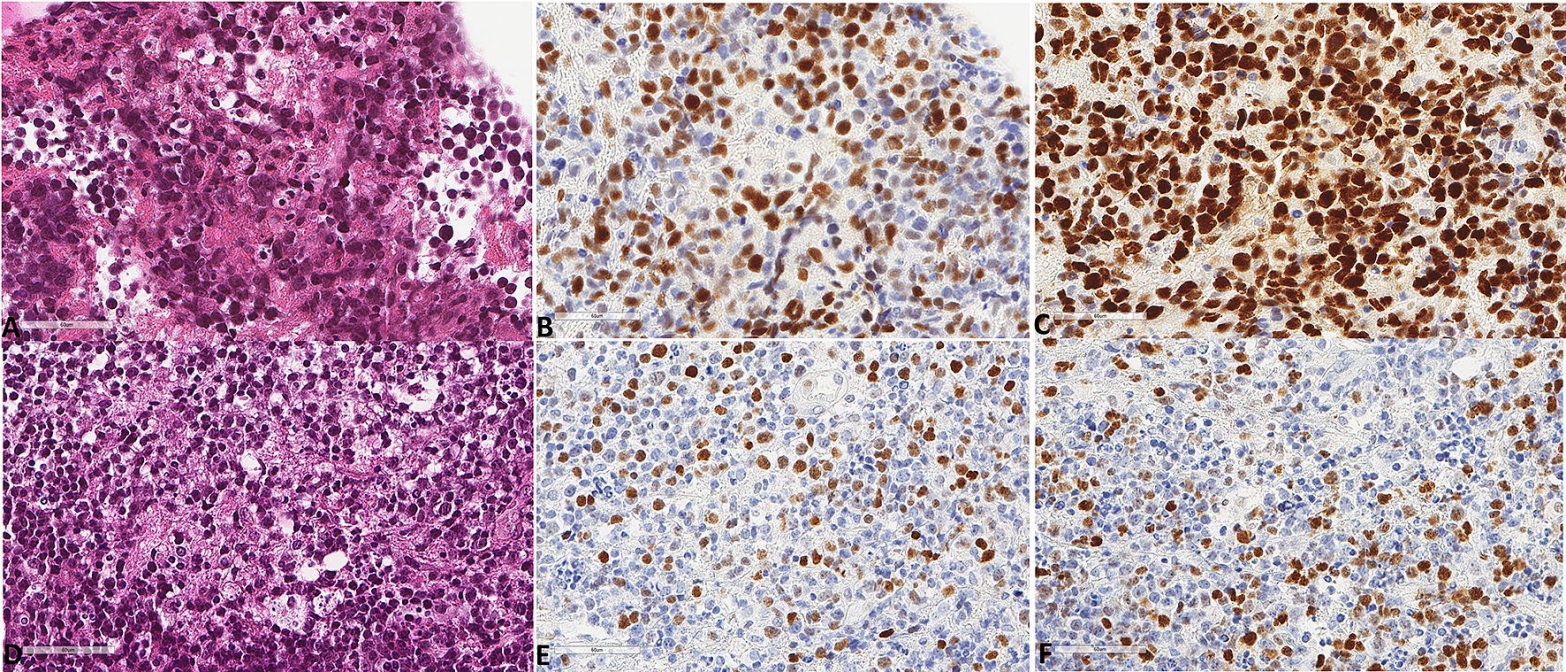
Representative microscopic images of two different PCNSLs diagnosed in two patients with MSKCC score C. All magnifications correspond to × 400 and represent the same focus between the morphological and the immunohistochemical images. (**A**) Hematoxylin, eosin, safran section. (**B**) MYC expression. (**C**) BCL6 expression. (**D**) Hematoxylin, eosin, safran section. (**E**) MYC expression. (**F**) BCL6 expression.

**Figure 2:**
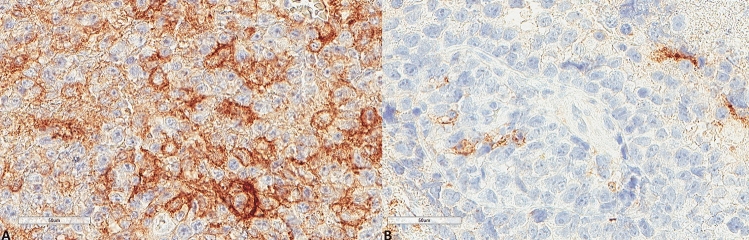
Representative images of PD-L1 expression. (**A**) Higher expression in a patient diagnosed with PCNSL MSKCC B group (× 400). (**B**) Lower expression in a patient diagnosed with PCNSL MSKCC C group (× 400).

Regarding the baseline characteristics of the lymphomas (Table 1), 70.8% of the tumors expressed MUM1, 65.2% expressed BCL2, and 72.7% expressed BCL6 (Fig. 1). Most cases (n = 18, 75%) were of the ABC type. All lymphomas were CD10 negative. Only three (12%) were double C-MYC/BCL2 co-expressors according to the aforementioned cut-off value. PD-L1 tumor cell expression (Fig. 2) was found in 19 tumors (76%); it showed a median of 10% (range 0–90) expression, with 9 tumors (36%) showing a positivity of at least 30% of tumor cells. These characteristics are in line with their reported frequency in previous studies^11,12,17^.

**Figure 3:**
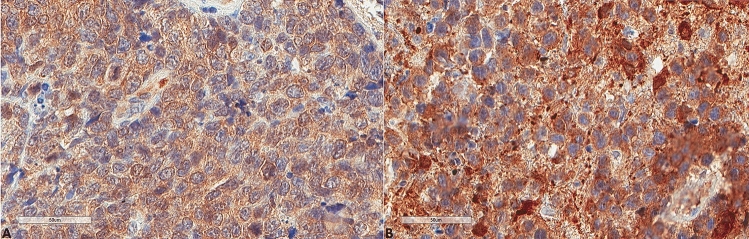
Representative images of LC3B expression. (**A**) Lower expression in a patient diagnosed with PCNSL MSKCC B group (× 400). (**B**) Higher expression in a patient diagnosed with PCNSL MSKCC C group (× 400).

**Figure 4:**
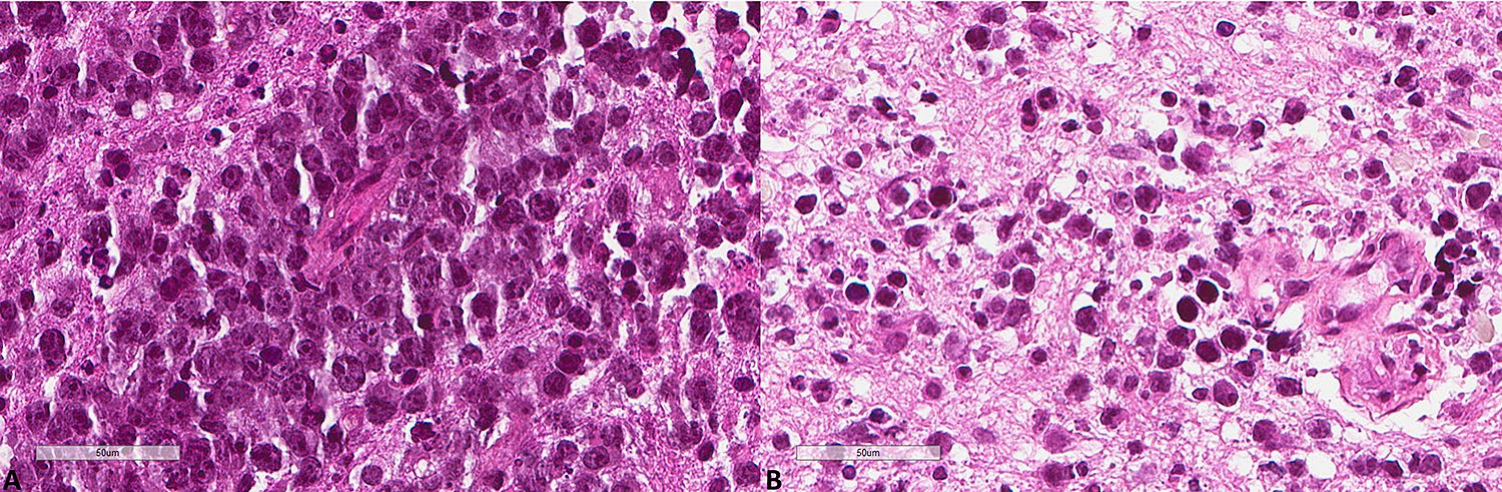
Representative morphological images. (**A**) Hematoxylin, eosin, safran section of the PCNSL shown in Fig. 3A (× 400). (**B**) Hematoxylin, eosin, safran section of the PCNSL shown in Fig. 3B (× 400).

**Figure 5:**
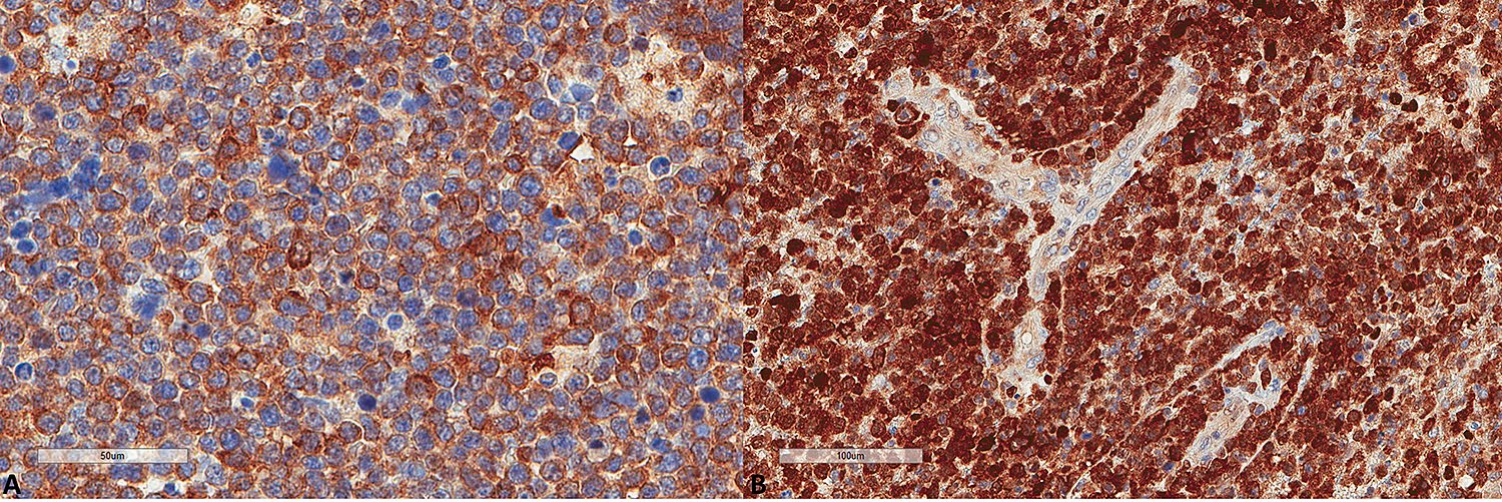
Representative images of p62 expression. (**A**) Lower p62 expression (× 400). (**B**) Higher p62 expression (× 400).

**Figure 6:**
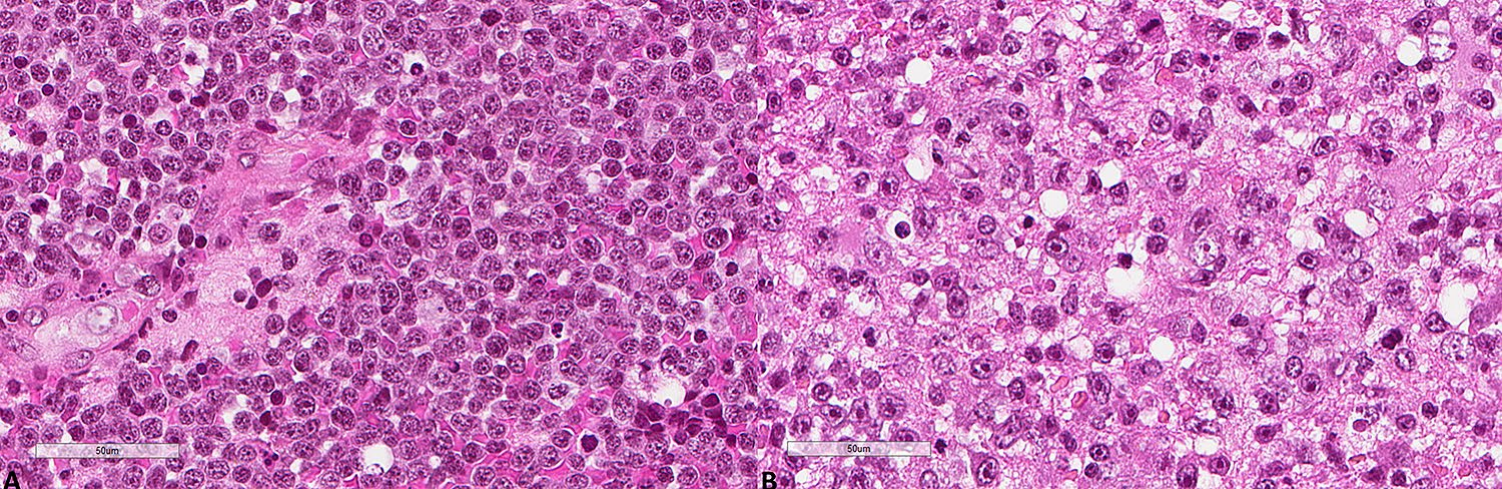
Representative morphological images. (**A**) Hematoxylin, eosin, safran section of the PCNSL shown in Fig. 5A (× 400). (**B**) Hematoxylin, eosin, safran section of the PCNSL shown in Fig. 5B (× 400).

**Figure 7:**
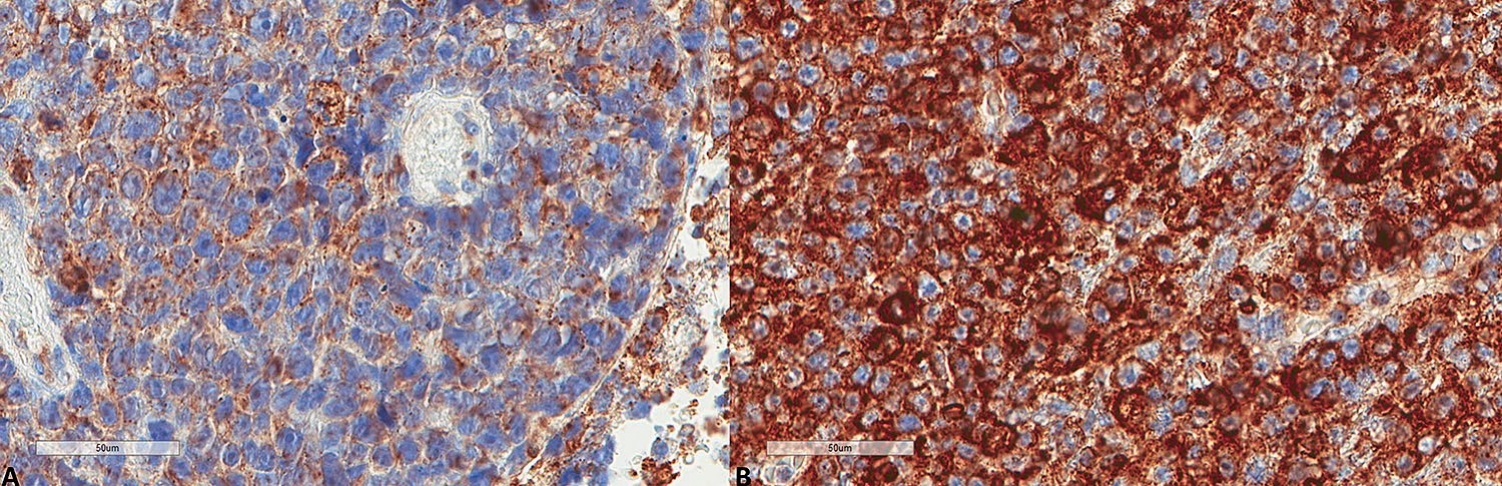
Representative images of M6PR expression. (**A**) Lower expression in a patient diagnosed with PCNSL MSKCC B group (× 400). (**B**) Higher expression in a patient diagnosed with PCNSL MSKCC C group (× 400).

The results for LC3B, p62, and M6PR expression are shown in Table 2. LC3B expression (Figs. 3, 4) was found in 15 (60%) tumors with a median H score of 30 (range 0–300) and a mean of 79.6 ± 102. Using the mean as a cut-off value, 14 (56%) lymphomas were of low expression, and 11 (44%) were of high expression. p62 expression (Figs. 5, 6) was present in most tumors (n = 21, 84%), being mild, moderate, and strong in 12%, 12%, and 60% of the cases, respectively. M6PR (Fig. 7) was present in all tumors, with H scores ranging from 10 to 300 (median 150) and a mean of 155.6 ± 110.4. Using this cut-off value, 14 (56%) cases were of low M6PR expression, and 11 (44%) were of high expression.

**Table 2:** Immunohistochemical findings of the autophagic pathway studied.

Parameter	N, %
**LC3B H score**	
Range	0–300
Mean	79.6 ± 102
Median	30
**LC3B group (cut-off: mean value)**	
Low	14, 56%
High	11, 44%
**p62**	
Negative	4, 16%
Mild	3, 12%
Moderate	3, 12%
Strong	15, 60%
**M6PR H score**	
Range	10–300
Mean	155.6 ± 110.4
Median	150
**M6PR group (cut-off: mean value)**	
Low	14, 56%
High	11, 44%
**Autophagic status group**	
Basal	14, 56%
High	3, 12%
Blocked	8, 32%

### Correlation of immunohistochemical findings with clinicopathological data (Table 3)

**Table 3:** Correlations of immunohistochemical findings.

	p62		p, x^2^	LC3B		p, x^2^	M6PR		p, x^2^	Group autophagy			p, x^2^
	High	Low		High	Low		High	Low		Basal	Blocked	High	
**Sex**													
Female	8	4	0.5, 0.4	8	4	**0.02**, 4.8	6	6	0.5, 0.3	4	7	1	**0.02, **7.3
Male	7	6		3	10		5	8		10	1	2	
**PS**													
≥ 70	6	6	0.3, 0.9	3	9	*0.06*, 3.3	5	7	0.8, 0.05	9	1	2	*0.05*, 5.9
< 70	9	4		8	5		6	7		5	7	1	
**MSKCC score**													
A	2	1	0.7, 0.6	1	2	*0.08*, 4.9	2	1	0.6, 0.7	2	0	1	*0.07*, 8.5
B	5	5		2	8		4	6		8	1	1	
C	8	4		8	4		5	7		4	7	1	
**Invasion** ^a^													
Yes	11	5	0.2, 1.4	7	9	0.9, 0.001	8	8	0.4, 0.6	9	5	2	0.9, 0.01
No	4	5		4	5		3	6		5	3	1	
**Multiplicity**													
Yes	5	4	0.6, 1.4	5	4	0.3, 0.7	3	6	0.4, 0.6	4	3	2	0.4, 1.5
No	10	6		6	10		8	8		10	5	1	
**MUM1**													
Yes	14	3	**0.0002**	8	9	0.8, 0.03	9	8	0.2, 1.1	9	8	0	**0.005**, 10.5
No	0	7	13.8	3	4		2	5		4	0	3	
**BCL2**													
Yes	9	6	0.6, 0.2	5	10	0.1, 1.8	7	8	0.3, 2.3	10	4	1	0.8, 0.02
No	4	4		5	3		4	4		3	3	2	
**BCL6**													
Yes	13	3	0.1	8	8	0.1, 2	7	9	0.7, 0.06	8	7	1	0.3, 2
No	3	3	2.1	1	5		3	3		5	1	0	
GC type	0	6	**0.004**	3	3	0.8	2	4	0.4	3	0	3	**0.02**
ABC type	14	4	12.8	8	10	0.05	9	9	0.5	10	8	0	11.6
Double^b^													
Yes	2	1	0.8, 0.06	0	3	0.1, 2.6	1	2	0.6, 0.1	3	0	0	0.2, 2.6
No	13	9		11	11		10	12		11	8	3	

Bold denotes statistical significance; italics denote statistical trend. MSKCC: Memorial Sloan Kettering Cancer Center.

^a^Invasion of deep brain structures.

^b^Double MYC/BCL2 co-expressors.

**Figure 8:**
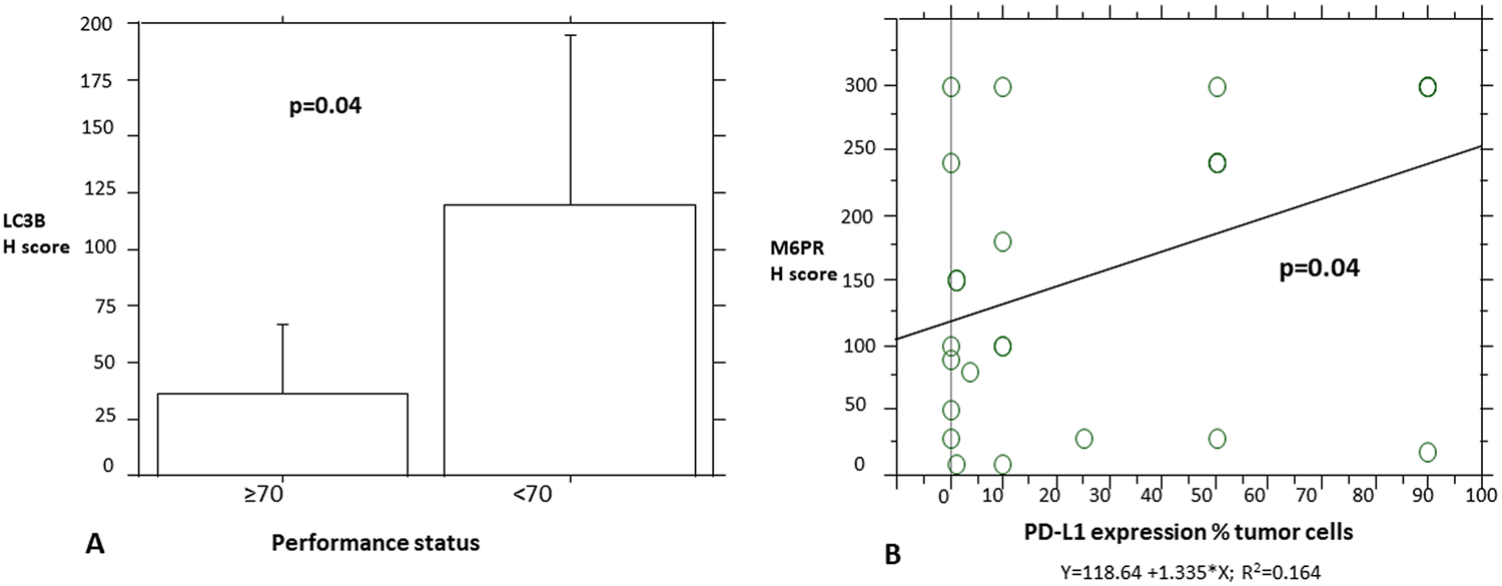
(**A**) The difference in LC3B H score between the two performance status groups. (**B**) Simple regression analysis comparing PD-L1 and M6PR expression.

The age at diagnosis was not found to be associated with the studied immunohistochemical factors by ANOVA analysis, followed by Fisher’s LSD (Least square difference) or by simple regression analysis. Only p62 expression showed a strong trend (Fisher’s LSD, p = 0.05) between the moderate and strong group expression levels with a mean age of 76 years vs. 60.4 years, respectively. Performance status was not significantly associated with the immunohistochemical factors studied, but it showed a trend of positive correlation with LC3B expression (p = 0.06, x^2^ = 3.3), since higher lymphoma LC3B expression was more often seen in patients with worse PS. This was further reflected in the autophagy groups (p = 0.05, x^2^ = 5.9), where most lymphomas from patients with PS < 70 were classified as displaying blocked autophagy. This was also reflected in the MSKCC score, where most highly LC3B-expressing tumors and blocked-autophagy-group tumors corresponded to group C patients (p = 0.08, x^2^ = 4.9 and p = 0.07, x^2^ = 8.5, respectively). The correlation of LC3B expression with the performance status (Fig. 8) was retained after ANOVA analysis for the H score, where we found mean LC3B H scores of 36.6 (± 46.9) for PS ≥ 70 and 119.2 (± 124.2) for PS < 70 (p = 0.04). A strong trend (p = 0.06) of association was also found with the MSKCC score after ANOVA analysis for the LC3B H score (A group, mean = 33 ± 49.3; B group, mean = 35 ± 47.1; C group, mean = 128.3 ± 125.1). No association was found with other clinical parameters, such as deep structure invasion, multiple lesions, complete response, recurrence after response, and the immunohistochemical factors studied.

**Figure 9:**
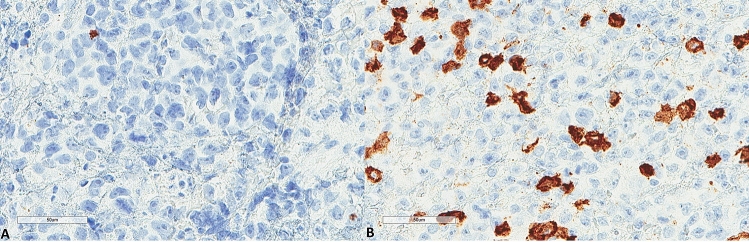
Representative images of CD8 expression. (**A**) Lower expression in a patient diagnosed with PCNSL MSKCC B group (× 400). (**B**) Higher expression in a patient diagnosed with PCNSL MSKCC C group (× 400).

Regarding the background immunohistochemical characteristics of the PCNSLs, p62 showed a strong positive association with MUM1 expression (p = 0.0005, x^2^ = 17.5), also reflected in the ABC subtype (p = 0.004, x^2^ = 12.8) and the autophagy groups, since non-MUM1-expressing tumors and GC tumors were associated with the high-autophagy group (p = 0.005, x^2^ = 10.5 for MUM1 expression and p = 0.002, x^2^ = 11.6 for GC subtypes, using the four-tiered system of p62 categorization). These features were not associated with LC3B expression (p = 0.8, x^2^ = 0.03 for MUM1 expression and p = 0.8, x^2^ = 0.05 for GC subtypes). Also, M6PR expression (Fig. 8) showed a positive correlation (p = 0.04, simple regression analysis) with PD-L1 expression and a trend (p = 0.08, x^2^ test) of positive association with CD8 T cell infiltration (Fig. 9). No association was found with p53, Ki67, CD8, BCL2, BCL6, or double co-expressors.

LC3B expression was not associated with p62 expression (p = 0.2, x^2^ = 1.3) or M6PR expression (p = 0.3, x^2^ = 0.8). M6PR expression was not associated with p62 expression (p = 0.7, x^2^ = 0.1).

**Figure 10:**
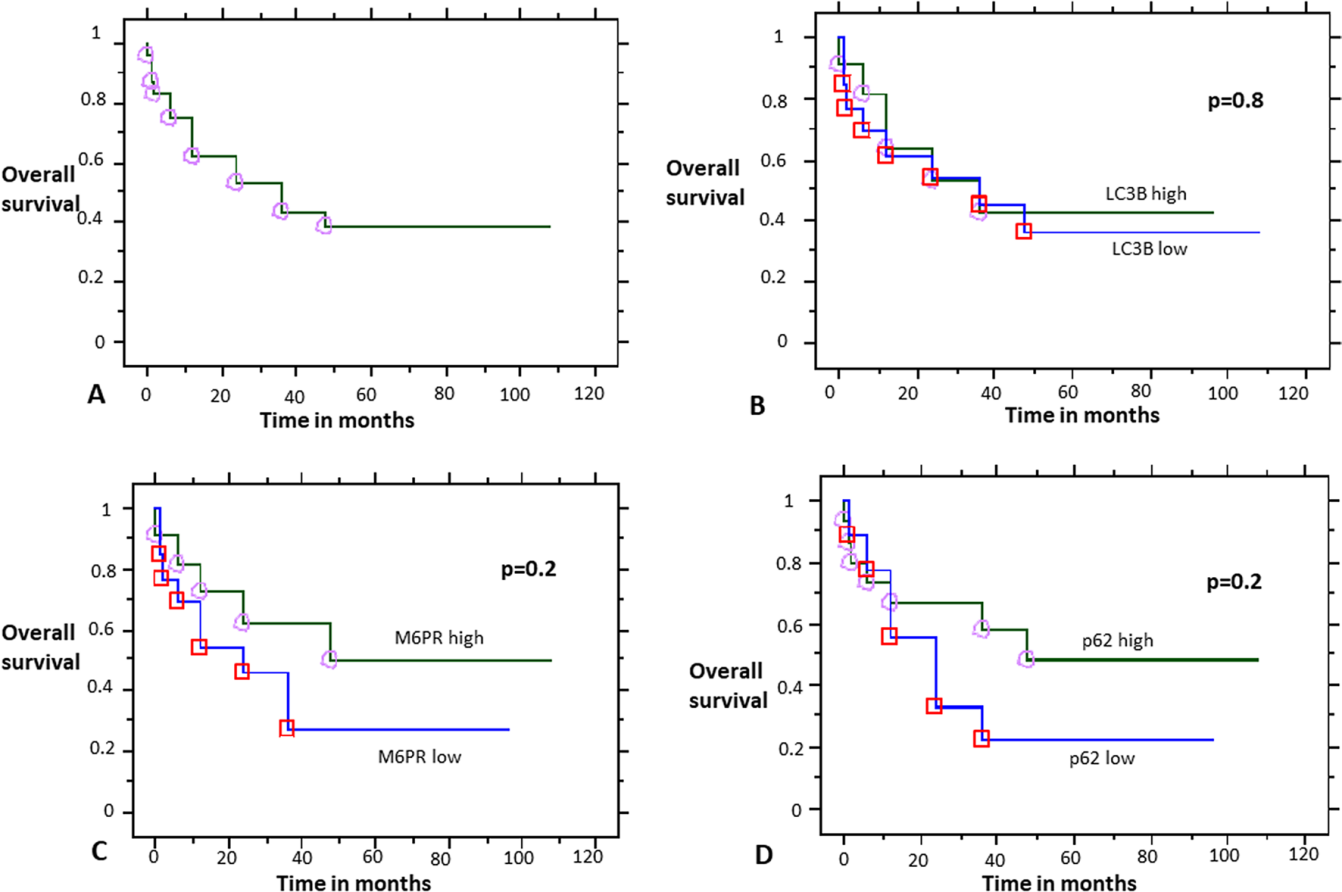
Survival analysis. (**A**) Overall survival analysis. Stratification by LC3B (**B**), M6PR (**C**), and p62 (**D**) expression.

Regarding survival analysis, despite higher M6PR and p62 expression showing better median survival of 48 vs. 24 months for the low-expression groups (Fig. 10), this did not reach statistical significance (p = 0.2 and p = 0.2, respectively). LC3B expression did not show prognostic significance (p = 0.8).

## Discussion

This is the first study, to the best of our knowledge, investigating the presence of autophagy in PCNSLs. Previous studies in systemic lymphomas have shown that higher expression of Beclin 1, a protein implicated in autophagy regulation, is associated with better prognosis in various forms of non-Hodgkin lymphomas^18^, including extranodal natural killer T-cell lymphoma^19^ and DLBCL^20^; the exact mechanism underlying this observation is unknown. By contrast, no correlation between Beclin-1 expression and survival was seen in multiple myeloma patients^21^. An increase in the quantity of autophagy-related proteins was also found in Hodgkin lymphoma cells to be associated with decreased expression of p62, suggesting activated autophagy and probably intact autophagic flux^22^. In the current study, we showed that almost half of the PCNSLs showed high LC3B expression. We also found that higher LC3B expression was associated with worse performance status and prognostic MKSCC score, implying a pro-tumoral role of autophagy in this tumor type. This would be in line with the cytotoxic effect of the pharmaceutical inhibition of autophagy in lymphoma cells: apilimod, an inhibitor of phosphatidylinositol-3-phosphate 5-kinase (PIKfyve) lipid kinase, an important regulator of endosome and lysosome function, showed maximal cytotoxic activity in malignant B cells^23^. The proposed mechanism for its action is that it disrupts the completion of autophagy, as indicated by an increase in p62 and LC3 in lymphoma cells^23^. This is considered to probably be provoked by impairment of endolysosomal membrane traffic, inhibiting the degradation of the autophagosomal cargo and finally leading to cellular stress and death^23^.

The use of p62 expression in conjunction with LC3 was previously suggested as a surrogate marker of activated autophagy, since the absence of p62 in the presence of LC3 would indicate degradation of p62 in autophagolysosomes and, thus, completion of the autophagic final step. However, p62 is also directly related to nuclear factor-kappa B (NF-kB) signaling. It is has been shown that NF-κB leads to p62 expression in chronic lymphocytic leukemia by controlling the expression of p62 mRNA^24^. Characteristically, most PCNSLs, in contrast to systemic DLBCLs, are characterized by increased NF-kB signaling mediated by frequent *MYD88* mutations (9). Thus, the strong p62 expression seen in the current series could also be related to NF-kB signaling in these tumors and not only to a blocked autophagy mechanism. In line with this hypothesis is the strong association of p62 with MUM1 found in the current series. Another link of NF-kB to autophagy has been noted in mantle cell lymphoma. TG2, an enzyme encoded by the TGM2 gene, a stress-responsive gene, is associated with NF-kB expression, and its up-regulation is correlated with poor prognosis in mantle cell lymphoma (MCL) patients^25^. Under stress, both TG2/NF-kB and their downstream IL-6 induce autophagy to promote MCL cell survival^25^. Additionally, NF-kB inhibition in lymphoma cells leads to reduced glucose availability, which triggers autophagy, which then prolongs cell survival, suggesting that the combined inhibition of NF-kB and autophagy is an option to achieve lymphoma cell death^26^.

It is interesting to note that lymphomas with MYC overexpression induce cytoprotective autophagy to escape stressful conditions^3^. Also, in BCL6-driven lymphomas, inhibition of autophagy through BCL6-mediated transcriptional repression of LITAF (lipopolysaccharide (LPS)-induced TNF alpha (TNFα) factor) has been proposed to be implicated in their pathogenesis^27^. However, in the current series, we did not find an association of the markers studied with MYC or BCL6 expression, probably explained by the limited number of GC-type PCNSLs.

As mentioned earlier, endosomal trafficking is also important in the autophagic machinery. Cell-surface receptors that facilitate the transport of their target proteins to lysosomes for degradation have been recognized, with the prototypical being the cation-independent mannose-6-phosphate receptor (CI-M6PR)^28^, an endosomal pathway marker associated with the autophagic machinery^6^. This receptor cycles constitutively between endosomes, the cell surface, and the Golgi complex and guides its cargos to the lysosomes, while M6PR is recycled^28^. This molecule relates chemotherapy and immunotherapy to autophagy^29,30^. Its upregulation on the tumor cell surface after chemotherapy augments T cytotoxic activity against tumor cells, thus enhancing chemotherapy and immunotherapy results^29–31^. Its upregulation is not related to de novo M6PR synthesis; rather, it is considered the result of increased M6PR transport from the cytoplasm to the cell surface, mediated by the autophagic process^29–31^. We investigated, for the first time, to the best of our knowledge, its expression in lymphomas, showing that it is present in most tumors and that its expression is associated with PD-L1 tumor cell expression and CD8 cytotoxic T cell infiltration; this suggests a possible role of immunomodulatory treatments in PCNSLs. Our results also add information on the immune microenvironment of brain tumors, confirming that, under certain conditions, this is not an immune-privileged compartment^13^.

The current study has certain limitations, such as its retrospective nature and the limited number of cases, both of which are difficult to avoid given the rarity of this disease. Furthermore, autophagy is a flux and should ideally be measured in functional assays^5^, because immunohistochemistry reveals the presence of autophagy constituents but not the whole system flow. Thus, other techniques that require fresh tissue or at least cell lines will be necessary to confirm these results. However, immunohistochemistry is the method of choice for tissue-based retrospective analysis^5^, and it at least provides a basis for a previously unexplored question, prompting further investigations.

Despite these limitations, this is the first study examining the expression of autophagy markers in PCNSLs, showing activated machinery in half of the tumors and p62 accumulation in most of them. Additionally, M6PR is a frequent finding in PCNSLs and is associated with the tumor immune microenvironment’s features. These findings provide evidence for the possible role of autophagy in PCNSLs and, thus, possible treatment targets.

### Compliance with ethical standards

The Local Ethics Committee of the University Hospital of Saint-Etienne, France (“Terre d’éthique”, Insitutional Review Board IORG0007394) approved the study (IRBN122021/CHUSTE); the acquisition of written informed consent was waived by the institutional review board given the retrospective nature of the study and the anonymization of all data. The study was performed according to the Declaration of Helsinki.

## Data Availability

Data are available upon reasonable request.
